# The dentato-rubro-thalamic tract as the potential common deep brain stimulation target for tremor of various origin: an observational case series

**DOI:** 10.1007/s00701-020-04248-2

**Published:** 2020-01-29

**Authors:** Volker Arnd Coenen, Bastian Sajonz, Thomas Prokop, Marco Reisert, Tobias Piroth, Horst Urbach, Carolin Jenkner, Peter Christoph Reinacher

**Affiliations:** 1 Department of Stereotactic and Functional Neurosurgery, Freiburg University Medical Center, Freiburg (i.Br.), Germany; 2 Faculty of Medicine, Freiburg University, Freiburg (i.Br.), Germany; 3 Brain Links/Brain Tools Cluster of Excellence, Freiburg University, Freiburg (i.Br.), Germany; 4 NeuroModul Basics (Center for Basics in NeuroModulation), Freiburg University, Freiburg (i.Br.), Germany; 5 Department of Neurology and Neurophysiology, Freiburg University Medical Center, Freiburg (i.Br.), Germany; 6 Department of Neuroradiology, Freiburg University Medical Center, Freiburg (i.Br.), Germany; 7 Clinical Trials Unit, Freiburg University Medical Center, Freiburg, Germany

**Keywords:** Deep brain stimulation, DRT, DRTT, Brain, Diffusion tensor magnetic resonance imaging, MRI, Parkinson’s disease, Tremor

## Abstract

**Introduction:**

Deep brain stimulation alleviates tremor of various origins. The dentato-rubro-thalamic tract (DRT) has been suspected as a common tremor-reducing structure. Statistical evidence has not been obtained. We here report the results of an uncontrolled case series of patients with refractory tremor who underwent deep brain stimulation under tractographic assistance.

**Methods:**

A total of 36 patients were enrolled (essential tremor (17), Parkinson’s tremor (8), multiple sclerosis (7), dystonic head tremor (3), tardive dystonia (1)) and received 62 DBS electrodes (26 bilateral; 10 unilateral). Preoperatively, diffusion tensor magnetic resonance imaging sequences were acquired together with high-resolution anatomical T1W and T2W sequences. The DRT was individually tracked and used as a direct thalamic or subthalamic target. Intraoperative tremor reduction was graded on a 4-point scale (0 = no tremor reduction to 3 = full tremor control) and recorded together with the current amplitude, respectively. Stimulation point coordinates were recorded and compared to DRT. The relation of the current amplitude needed to reduce tremor was expressed as TiCR (tremor improvement per current ratio).

**Results:**

Stimulation points of 241 were available for analysis. A total of 68 trajectories were tested (62 dB leads, 1.1 trajectories tested per implanted lead). Tremor improvement was significantly decreasing (*p* < 0.01) if the distance to both the border and the center of the DRT was increasing. On the initial trajectory, 56 leads (90.3%) were finally placed. Long-term outcomes were not part of this analysis.

**Discussion:**

Tremor of various origins was acutely alleviated at different points along the DRT fiber tract (above and below the MCP plane) despite different tremor diseases. DRT is potentially a common tremor-reducing structure. Individual targeting helps to reduce brain penetrating tracts. TiCR characterizes stimulation efficacy and might help to identify an optimal stimulation point.

## Introduction

Deep brain stimulation is a potent electrical stimulation therapy to treat medically refractory tremor of various origins. Typically, the thalamic target region and more specific the ventral intermediate nucleus (Vim) are used to treat tremor in tremor-dominant Parkinson’s disease (thus without hypokinetic symptoms) and essential tremor (ET) [6, 7, 30, 31, 40, 61]. In ET it has become routine to target more inferior regions (below the mid-commissural point plane), aiming for the caudal zona incerta (cZI) [46, 47, 62] or the subthalamic area (STA) [27, 33, 48]. We will here jointly regard cZi and STA as “subthalamic region” (STR).

With the introduction of diffusion tensor imaging (DTI)-based approaches in DBS planning, the dentato-rubro-thalamic tract (DRT sometimes DRTT in the literature) was identified as a potential target structure for tremor [13–15]. Moreover, it was hypothesized that the mentioned target regions (Vim, STR, cZI) are actually confluent with the DRT which is hit in different (thalamic and subthalamic) parts (“three stereotactic targets, one fiber pathway” concept) [13, 14]. Several groups have since used DTI tractography to modulate the DRT in DBS surgery [3, 19, 21, 57, 60] and have meticulously refined targeting strategies with good results [43]. Moreover, evidence for DRT as being a marker structure for the identification of the Vim at MCP level has been reported on stimulation grounds [14, 57] and based on related intraoperative electrophysiology [29]. For an overview on the topic, cf. [9]. Recently, the use of DTI-assisted targeting of the DRT for focused ultrasound lesioning was also described [11]. Beyond clinical effects, the claim that the DRT might be the actual common tremor-reducing structure for several indications has not been proven so far [23].

**Fig. 1:**
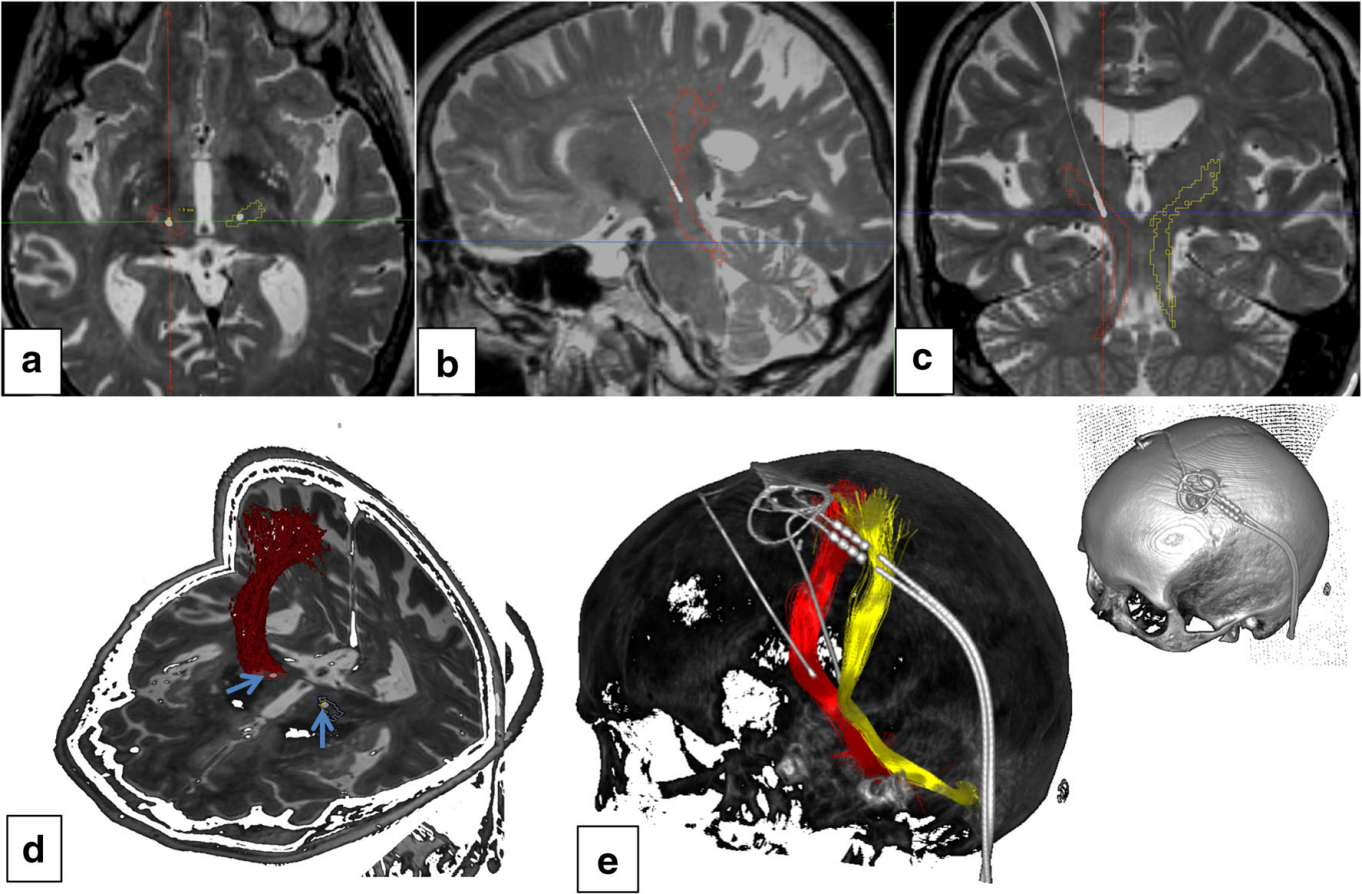
Clinical example of typical Vim/DRT DBS. **a**–**c** Axial, sagittal, and coronal depiction, respectively. T2-weighted MRI, electrodes superimposed from helical CT. **d** Quasi three-dimensional view from anterior superior and left. Blue arrows indicate DBS electrodes. **e** Three-dimensional representation of the tractography result (DRT: left = yellow; right = red)

The present study was designed to find first evidence for the hypothesis of tremor-reducing potency of direct DRT stimulation in several clinical indications (tremor-dominant Parkinson’s disease, essential tremor, dystonic tremor, tremor in multiple sclerosis) and was geared to estimate an effect size for future studies. The subthalamic and thalamic portions (cf. Fig. 1) of the DRT were targeted with DTI tractographic methods according to clinical indications. We used detailed protocols from intraoperative acute stimulation effects in 36 patients undergoing DBS surgery and a combination of pre- and postoperative imaging to retrospectively evaluate the *symptom improvement to distance relationship* for the DRT. We have introduced a new efficacy measure (TiCR = tremor improvement per current ratio) for a better appreciation of this relationship. Long-term clinical tremor outcomes were not part of this evaluation. TiCR is a simple efficacy measure that is introduced in the present study as an efficiency measure for DBS tremor surgery. It represents the tremor improvement on a 4-point scale (0–3; 0 being no improvement, 3 being 100% tremor control) divided by the current applied (in milliampere = mA and as used here in 0.5 mA increments) and allows to draw conclusions regarding the symptom improvement to distance relation.

## Methods

Patients with various diagnoses were selected for thalamic (Vim/DRT) or subthalamic region (STR/DRT) DBS surgery because of disabling and treatment refractory tremor. Decision for surgery was made interdisciplinary after failure of medical therapy according to standardized guidelines from German Society of Neurology (www.dgn.de). Patients were operated between 2010 and 2016. Patients were included if detailed intraoperative records as well as adequate imaging were available for retrospective evaluation. The study was approved by the local IRB (Freiburg University, No. 567/14). As a standard, we perform DTI studies in all patients undergoing DBS surgery.

*Imaging studies* were performed on clinical MRI systems (Siemens Magnetom Trio Tim System 3 T, Erlangen, Germany) or Philips Intera 3 T (Philips Healthcare, Best, Netherlands).

### Siemens

Anatomical sequences: 12-channel head coil. 3-D MPRAGE (magnetization-prepared rapid gradient echo): TR 390 ms, TE 2.15 ms, TI 800 ms, flip angle 15°, voxel size 1.0 × 1.0 × 1.0 mm3, acquisition time 3:15 min. 3-D T2 SPACE-sequence: TR 2500 ms, TE 231 ms, echo train length 141, flip angle variable, voxel size 1.0 × 1.0 × 1.0 mm3, acquisition time 6:42. Diffusion tensor imaging: single-shot 2-D SE EPI, TR 10,000 ms, TE 94 ms, diffusion values *b* = 0 s/ mm2, *b* = 1000 s/mm2, diffusions directions 61, slice count 69, voxel size 2.0 × 2.0 × 2.0 mm3, acquisition time 11:40. Deformation correction of the EPI sequence according to Zaitsev et al. 2004 [63].

### Philips

An isotropic T2-weighted fast spin-echo sequence, a DTI sequence, and 2 magnetization-prepared rapid gradient echo scans, using an 8-element phased-array head coil, were acquired. The parameters were the following: fast spin-echo, repetition time (TR) = 12.650 ms, echo time (TE) = 100 ms, field of view (FOV) = 254 mm, matrix = 176 · 176, 120 sections, sections thickness = 1.44 mm, and acquisition time = 3 min and 44 s. The resulting data were reconstructed to isotropic (1.44 · 1.44 · 1.44)-mm^3^ voxels. For DTI a single-shot spin-echo echo planar imaging pulse sequence with TR = 13.188 ms,TE = 84 ms,FOV = 256 mm,matrix = 128·128, 70 sections, section thickness = 2 mm, number of gradient directions = 32, b-value = 1000 s/mm^2^, sensitivity encoding factor 2.9, acquisition time = 7 min 54 s with isotropic reconstructed (2 · 2 · 2) mm^3^ voxels was acquired. A T1-weighted 3-D magnetization-prepared rapid gradient echo sequence was acquired before (structural information) and after (vessel visualization) contrast administration (gadolinium-diethylenetriamine pentaacetic acid) with a sensitivity encoding factor = 4, TR = 8.5 ms, TE = 3.8 ms, flip angle = 8°, FOV = 256 mm, matrix = 256 · 256, 160 sections, section thickness = 2 mm, and acquisition time = 4 min 17 s. This resulted in reconstructed isotropic (1 · 1 · 1)-mm^3^ voxels. All images were obtained in axial orientation.

### DTI FT-assisted planning

The tractographic reconstruction of the individual DRT has been previously described [13, 15, 16]. In brief we performed DTI FT on a Stealth Viz DTI system (Medtronic, USA) using the midbrain as initial seed regions (the primary motor cortex and the *dentate nucleus of the ipsilateral cerebellum* as inclusion ROI). Trajectory planning was performed on a FrameLink system (V5.0, Medtronic, USA). In the antero-posterior direction (AP), coordinates were first chosen for 1/4 to 1/3 of the distance from PC to AC and then adapted according to tractographic DRT rendition. For laterality we started with 10 mm from third ventricular wall before adapting with tractographic imaging.

**Fig. 2:**
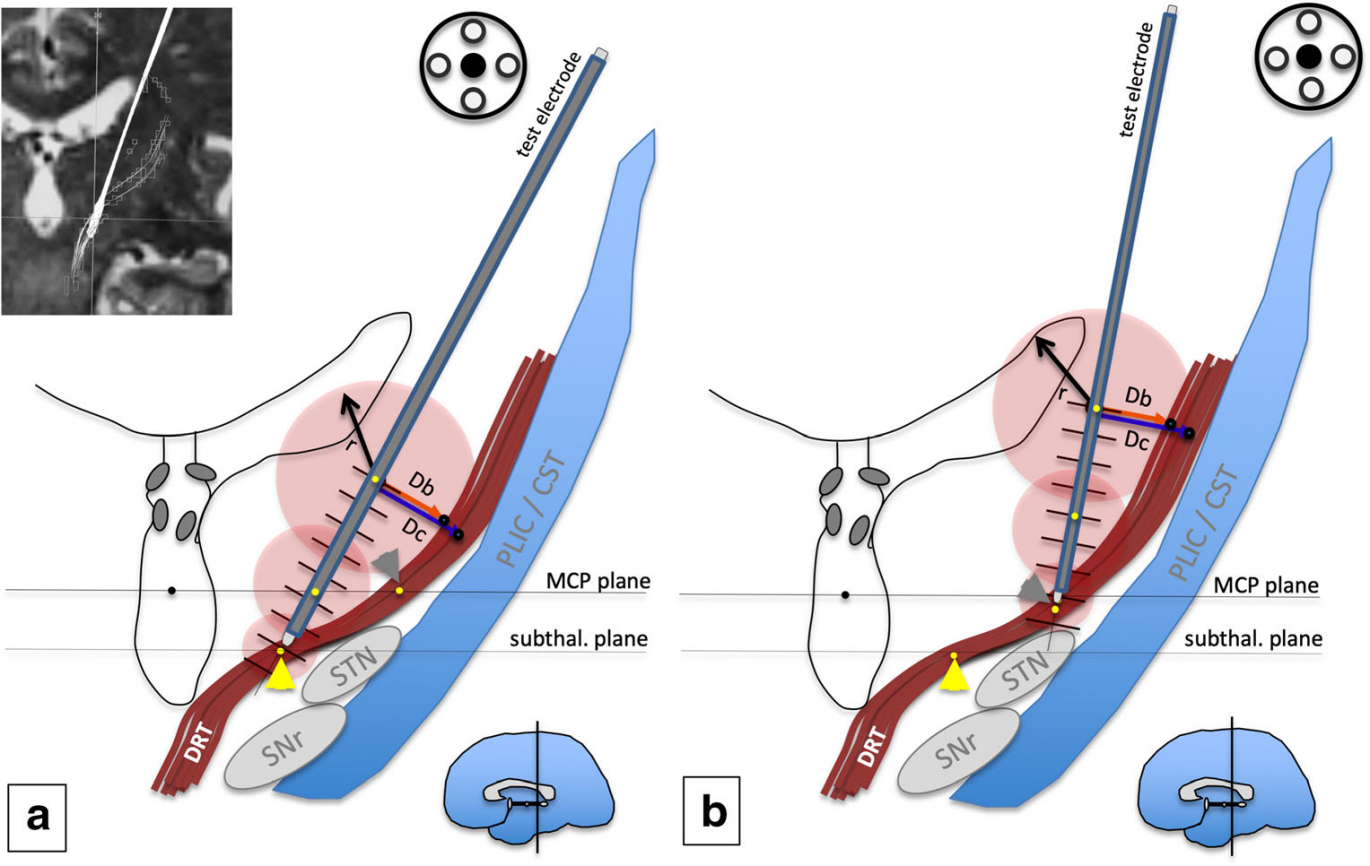
Experimental setup. **a** Targeting the subthalamic region (STR, yellow arrow head). The target region is located well below the MCP plane in the quasi-horizontal part of the DRT (crossing above the STN). **b** Targeting the traditional thalamic Vim (ventral intermediate nucleus, gray arrowhead in a) target. The test electrode (gray) is lowered into the target region in increments of 2 mm. Electrodes are here in the target position (“0”). Tremor response is recorded at each stimulation point together with the current needed. This current decreases as the target proximity increases (indicated by decreasing diameter of the electric fields, red). MCP coordinates of stimulation points are recorded along the trajectory (yellow points). From this stimulation point, the closest distance to the DRT (border and center) is recorded as a line (Db, Dc). The MCP coordinates of the closest DRT border point and the closest DRT center point are then recorded. *MCP* mid-commissural point, *STN* subthalamic nucleus, *SNr* subtstantia nigra, *PLIC* posterior limb of internal capsule, *CST* cortico-spinal tract

According to our previous results [13, 14], we targeted the anterior two third of the visualized DRT in thalamic (cf. Fig. 2b) and subthalamic regions. In the latter, we aimed for the most horizontal part just medial to the posterior STN (cf. Fig. 2a). Individual asymmetries of the DRT were taken into account and trajectories adjusted in order to reach the DRT, leading sometimes to asymmetric trajectories. Safe trajectories entered pre-coronally and avoided sulci, ventricles, and vessels visible on T1-weighted contrast-enhanced MRI. We and others interpret the literature and find that DRT penetrates the thalamic level at the region where the Vim(VLa/VLp) nuclei are located. However, we think that different diseases demand different implantation strategies. For example, a patient with Parkinson’s tremor should not be stimulated far below the MCP level because of a tendency to increase axial instability. Therefore, in these cases, we chose the DRT but at the MCP level (cf. Fig. 2).

*Stereotactic surgery* was performed with the patient under local anesthesia and analgo-sedation with remifentanil (Ultiva®, GlaxoSmithKline, London, UK) when necessary. Patients were fully awake for acute testing. A Leksell G-Frame (Elekta, Sweden) was mounted on the patient’s head. A planning CT was performed with a localizer box (Elekta, Sweden). After fusion of the imaging data (including T1W and T2W high-resolution anatomical sequences and dFT depiction of the DRT) and determination of the stereotactic coordinates (FrameLink, V5.0, Medtronic, USA), the test electrodes (Cosman, Burlington, Massachusetts; 2 mm exposed tip, 1.3 mm diameter) were lowered into the target region after a coronal burr hole was made and the dura opened. Intraoperative testing occurred with a stimulation device (RFG 1A, Cosman, USA). Electrodes were guided with a microdrive (FHC, Bowdoin, Maine) in principle allowing 2-mm steps anterior, lateral, medial, and posterior and sub-millimeter steps along the trajectory. DBS electrodes (model 3389, Medtronic, USA) were implanted under fluoroscopic guidance and fixed in burr-hole caps. Internal neural stimulators (ACTIVA series, Medtronic, USA) were placed subcutaneously beneath the clavicle or abdominally under general anesthesia in the same session. In a single case, a Vercise RC system (Boston Scientific, Valencia, USA) with an octopolar electrode was implanted.

### Intraoperative test stimulation

Stimulation was performed on the initial trajectory (planned, center). Only if side effects at an unexpected threshold occurred or if tremor improvement was found to be too low, a second trajectory was chosen. The target region was tested typically beginning on the trajectory minimal 10 mm (typically 8 mm) superficial to the planned target and ended at max 6 mm (typically 2 mm) inferior to it. Not all possible stimulation points were used (see limitations, Table 2). Stimulation was performed with monopolar stimulation (frequency, 150 Hz; pulse width, 100 μs; electric current, 1–7 mA) starting with 0.5 mA in 0.5-mA increments. Tremor reduction and side effects (not reported here) were examined. In our clinical setting, raters were intraoperatively not entirely blinded for electrode position or stimulation depths and intensity (see limitations). Tremor was graded on a rating scale by a neurologist experienced in movement disorder surgery and graded between “0” (no effect on tremor) to “3” (full tremor arrest). A tremor effect of “2” was given for a reduction by 50% (either severity or amplitude). The position of individual stimulation points on the trajectory together with the applied current was noted in a standardized protocol for postoperative evaluation (cf. Fig. 2). Stimulation was not performed on any other than the central electrode path if stimulation effect was judged to be favorable on this first pass.

### Postoperative acquisition of stimulation points

Postoperative helical computed tomography (CT) was fused to preoperative planning in the FrameLink software (Medtronic, USA) including the DTI rendition of the DRT. The MCP system had been defined based on MRI. Visualization of the final DBS electrode was used as a reference to simulate intraoperative test electrode positions (final implantation depth as determined with intraoperative fluoroscopy was taken into account). If a different path (e.g., anterior) had been used during surgery, a respective tract parallel to the final DBS electrode was defined. MCP coordinates of the intraoperative stimulation points were simulated and recorded. From each individual stimulation point, a measurement of the Euclidian shortest distance to the border of the DRT (Db) and the center of the DRT (Dc) was performed in the correlated triplanar display (axial, coronal, sagittal) and additionally perpendicular views to the trajectory (*probe’s eye view*). Distance to (in mm) and actual MCP coordinates of DRT border and DRT center were recorded (cf. Fig. 2).

### Statistics

The influence of “distance to border of DRT,” “distance to center of DRT,” and distance on trajectory on TiCR and tremor improvement was analyzed using a linear mixed model with variance components covariance structure and restricted maximum likelihood estimation including “distance to border of DRT” (distance to center of DRT, respectively) as fixed effect and “patient number” as random effect to control between subject variations. Thus, random intercepts are estimated per patient.

Binary endpoint analysis was used for the entire population. As an endpoint, Ti (tremor improvement) was categorized as responder (Ti > =2) vs. nonresponder (Ti < 2). Analysis was done using a mixed effects logistic regression with variance components covariance structure and restricted maximum likelihood estimation including “distance to border” as fixed effect and “patient number” as random effect to control for between subject variations.

### Atlas visualization

**Fig. 3:**
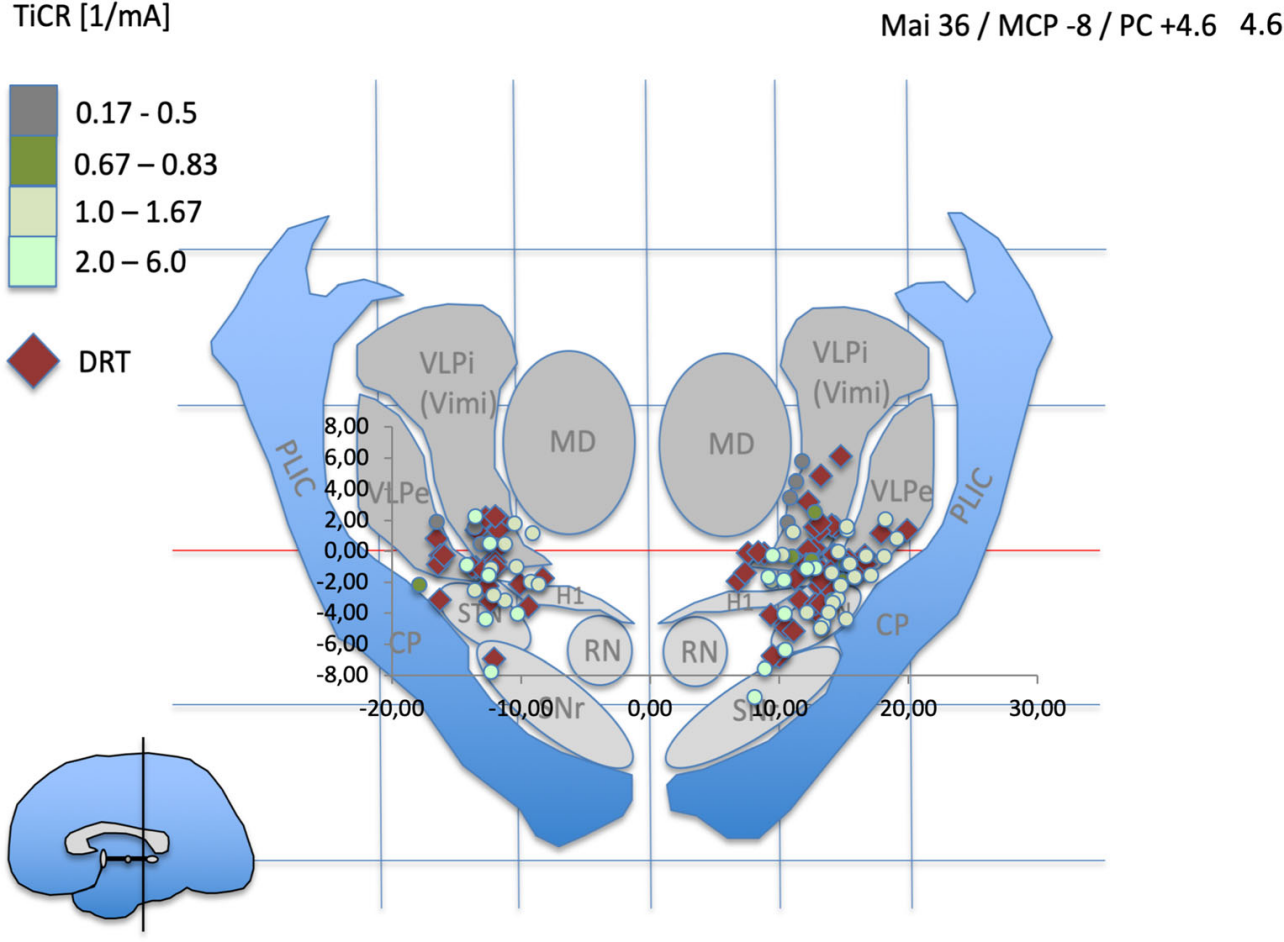
Atlas representation of stimulation points (colored dots) and depiction of DRT center (red diamonds) in the mid-commissural point (MCP) system. Normalized data (see Methods section). Idealized atlas slices after Mai et al. (Atlas of the human Brain, 3rd edition, Academic press, 2007^4^). PC + 4.6 is just anterior to the DRT. Light green dots indicate most efficient tremor reduction at minimal current expense as expressed by TiCR. Stimulation coordinates (differentially colored dots) and DRT coordinates are paired. Pairing is not shown for better visualization

**Fig. 4:**
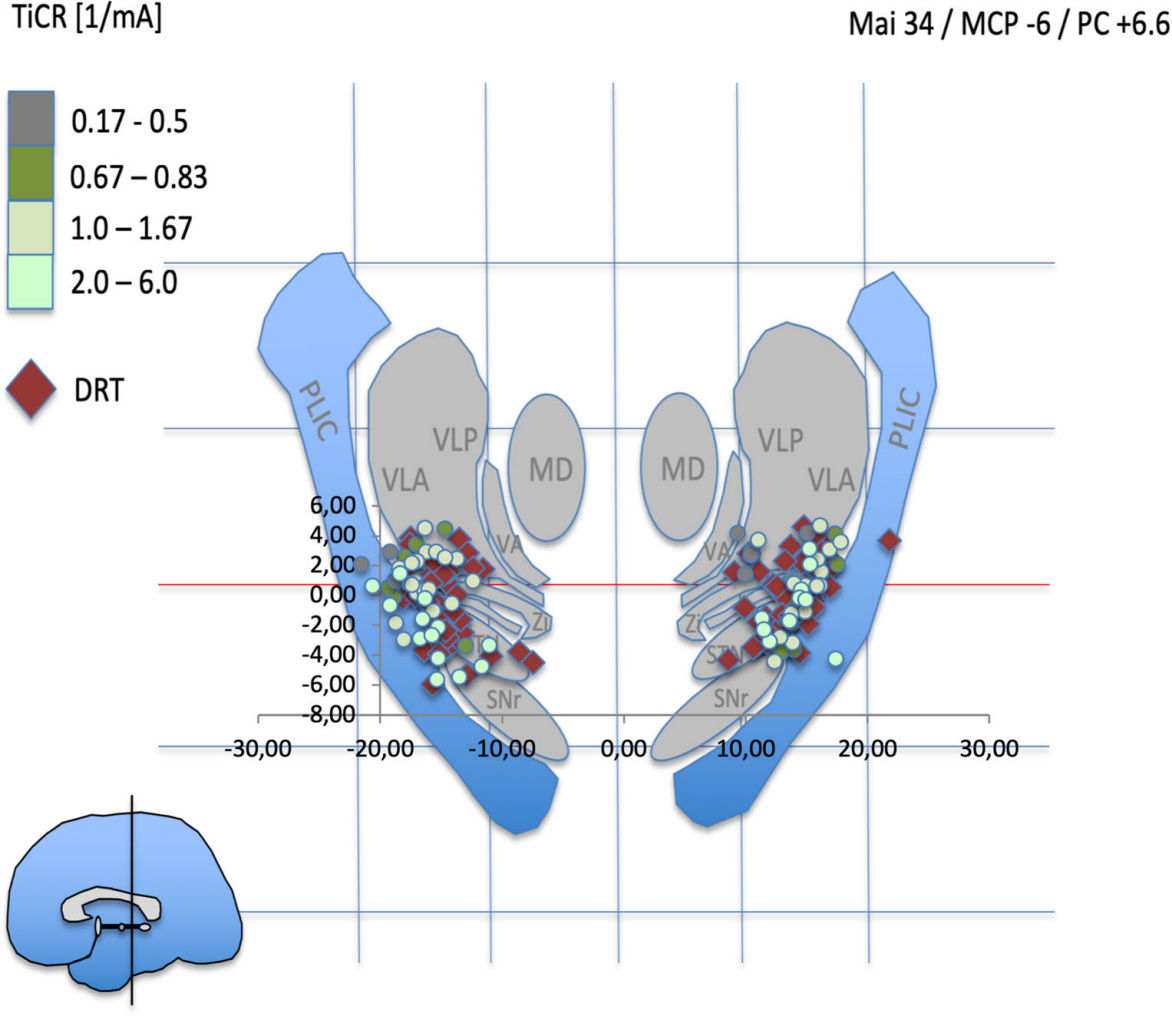
PC + 6.6 represents the typical stimulation region in thalamic (Vim) DBS. Note how DRT center points and stimulation points with excellent TiCR focus on the region between lateral STN and VLA

**Fig. 5:**
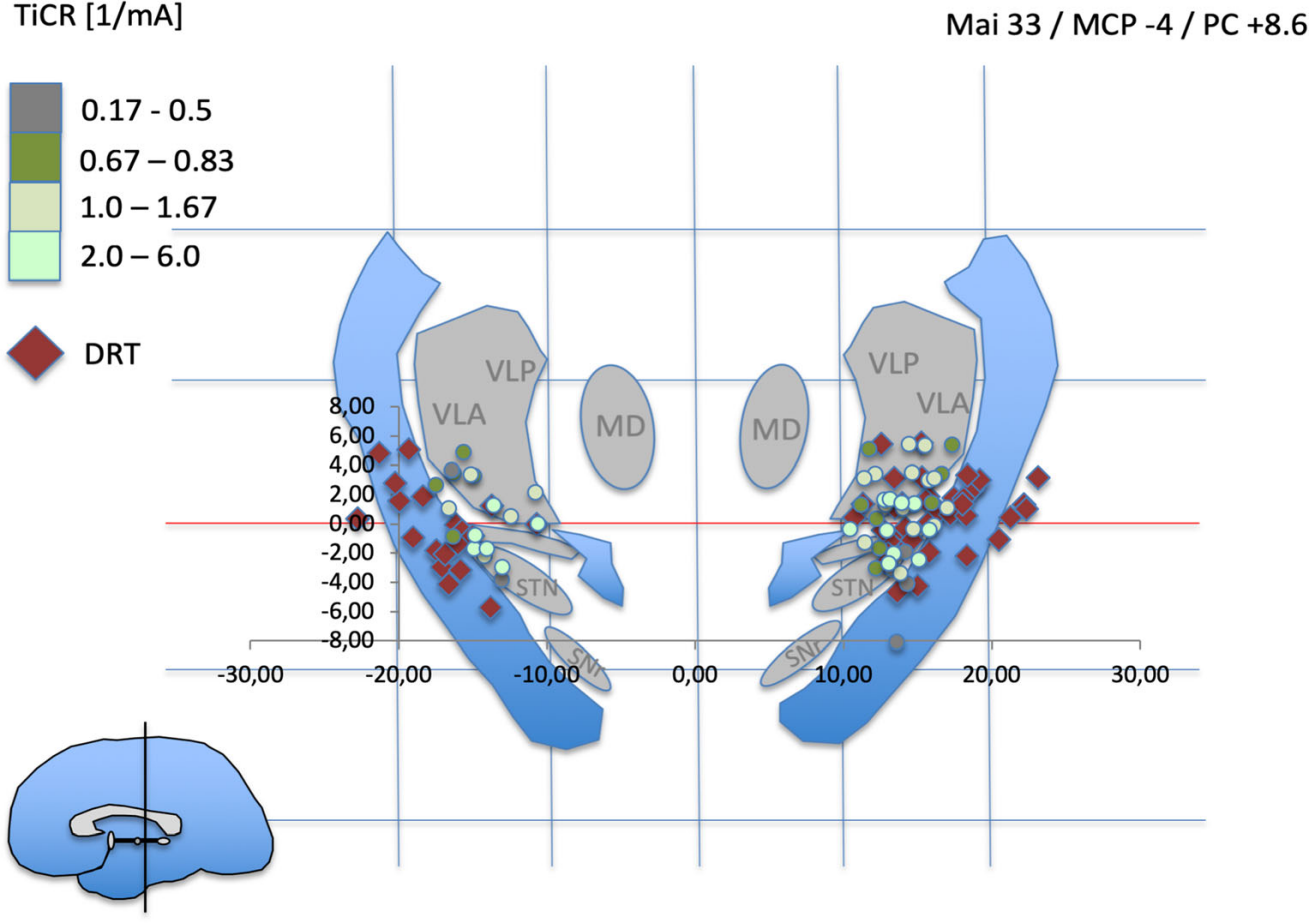
PC + 8.6 mm shows a further concentration of beneficial stimulation points (light green) on the DRT. *STN* subthalamic nucleus, *SNr* substantia nigra, *RN* red nucleus, *CP* cerebral peduncle, *PLIC* posterior limb of internal capsule, *H1* Forel’s field H1, *MD* mediodorsal nucleus of thalamus, *VLA* ventrolateral anterior nucleus, *VLP* ventrolateral posterior nucleus, *VLPi* ventrolateral posterior internus, *VLPe* externus nucleus, *Zi* zona incerta

In order to visualize the stimulation points, MCP coordinates (x, y, z) were divided by the individual ACPC distance and multiplied with the ACPC distance of the Mai atlas [35]. Three exemplary coronal templates were identified and idealized, and the stimulation points were plotted (cf. Figs. 3, 4, and 5).

## Results

A total of 36 patients (64 ± 13.6 years, 17 female) were enrolled. Diagnoses were essential tremor (*n* = 17, 70.6 ± 6.4 years), Parkinson’s tremor (*n* = 8, 74.4 ± 3.9 years), multiple sclerosis (*n* = 7, 45 ± 7.2 years), dystonic head tremor (*n* = 3, 45 ± 7.2 years), tardive dystonia, and tremor (*n* = 1, 64 years).

In 18 patients, the classical ventral intermediate nucleus (Vim) region was directly targeted with DRT visualization. In the remaining 18, a subthalamic target (STR = subthalamic region) was chosen, and in both instances, trajectories were planned with tractographic DRT visualization.

The mid-commissural point (MCP) coordinates of the tractographically planned targets are presented in Table 1. On inspection these coordinates do not deviate from expected ones especially for the Vim target (17).

**Table 1:** Mid-commissural point (MCP) coordinates of distinct target regions

	**STR/L**			**STR/R**		
	**X**	**Y**	**Z**	**X**	**Y**	**Z**
**Mean**	12.3	− 7.8	− 4	11.8	− 5.8	− 3.9
**Stddev**	1.8	3	1.8	2.1	3	2.1
**Maximum**	15.8	2	0	16	2.5	0
**Minimum**	9.9	− 12.1	− 7.2	9	− 9.4	− 8.7
	**Vim/L**			**Vim/R**		
	**X**	**Y**	**Z**	**X**	**Y**	**Z**
**Mean**	12.7	− 6.5	− 0.8	11.4	− 6.3	− 0.6
**Stddev**	2.1	1.7	1.4	1.9	1.6	1.4
**Maximum**	16.6	− 4	1.3	15.2	− 3.6	2.9
**Minimum**	10	− 9.8	− 3	7.7	− 8.7	− 2.8

*STR* subthalamic region, *Vim* ventral intermediate nucleus of thalamus

Negative Y indicates posterior MCP; negative Z indicates inferior to MCP

In 62 electrode placements, 68 trajectories were tested intraoperatively (1.1 trajectories tested per implanted DBS electrode). Based on results of intraoperative test stimulation (side effects, mainly paresthesia), 56 out of 62 electrodes (90.3%) were finally implanted on the initially planned trajectory (9.7% deviation from initial planning). Overall, 251 stimulation point data sets were acquired of which full data for analyses was available for 241 points (the uneven distribution of stimulation points over complete trajectories – due to retrospective nature – is detailed in Table 2).

**Table 2:** Stimulation point distribution over entire trajectories (− 6 = below target to + 10 mm above target)

On trajectory distance to target	Total	Missing	Total valid	Mean	Std
− 6	1	0	1	1.5	.
− 4	1	0	1	0.8	.
− 2	15	1	14	1.2	0.7
***0***	***57***	***5***	***52***	***2.0***	***1.2***
1	1	0	1	1.0	.
2	52	0	52	1.6	1.3
4	56	1	55	1.4	1.1
6	54	4	50	1.1	0.9
8	14	0	14	1.2	1.5
10	1	0	1	1.0	.
**Total**	**252**	**11**	**241**	**1.5**	**1.2**

Note that due to retrospective study design, positions 8, 6, 4, 2, 0 and − 2 were more frequently tested than other positions

The atlas visualization revealed a coincidence of most beneficial stimulation points (TiCR > 2/mA) with expected thalamic nuclei and subthalamic target regions (e.g., VLA/VLP nuclei; cZi). These results are congruent with human anatomical work [24, 26, 42]. Overall, beneficial stimulation points showed close proximity to DRT-related points (center of DRT, cf. Figs. 4 and 5). In more inferior – subthalamic – positions, more medial stimulation points occur with better TiCR. TiCR proves to be a beneficial measure to appreciate the efficacy of stimulation to distance relationship. The ACPC-based normalization is likely not optimal for the medial-lateral gradient (cf. Figs. 4/3b, 5/3c ). Atlas representation is not intended as analysis but simply serves to check visual plausibility.

### Statistics

**Fig. 6:**
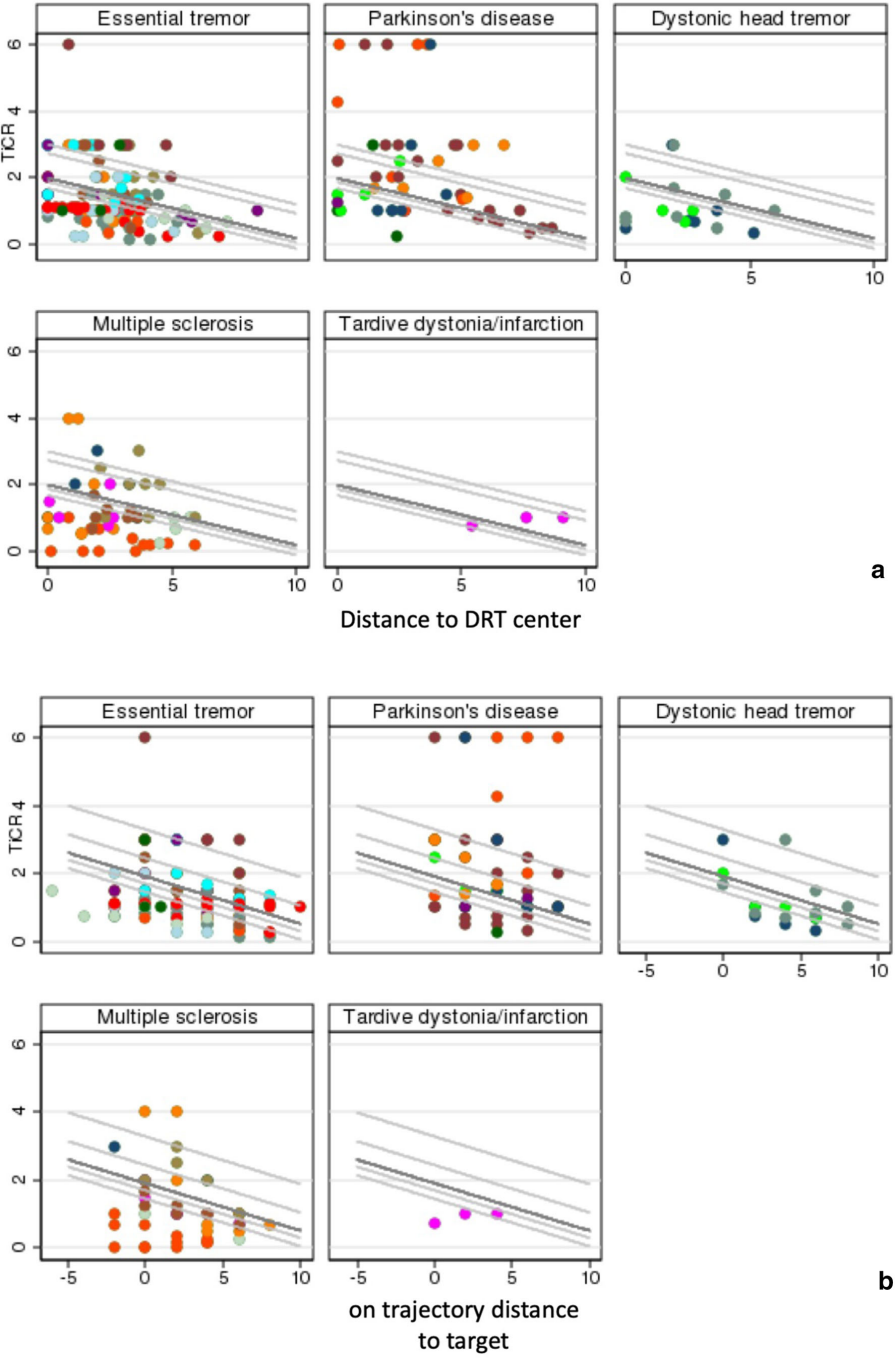
Observed data of the distance to DRT (**a**) and the distance on the trajectory (**b**) when compared to TiCR. In dark gray, the estimated LMM is added (beta = − 0.18, fixed intercept = 1.98). Exemplarily, some random effect lines were added to illustrate the performed analysis. The graphs are separated by diagnosis, for illustrational purpose. The analysis, however, was performed over all patients. Therefore, the resulting line and its respective slope are the same in each graph

The outcome measure TiCR was significantly decreasing (*p* < 0.01) if the distance to both the border and the center of the DRT was increasing. The estimate is interpreted as an effect of − 0.13 (St.Err. 0.05,) change in TiCR per 1-mm change in distance to border of DRT (deviance of linear mixed model: 717.8). For distance to the center of the DRT, the effect estimate was − 0.18(St.Err. 0.04) change in TiCR per 1-mm change (deviance of linear mixed model: 711.5), respectively. Adding a random patient effect in the regression model meant that in the analysis, it was considered that different patients might have different (baseline) levels of response, but the overall effect of shortening the distance to the border to the DRT is the same in every patient (cf. Fig. 6). Figure 6a and b show the observed data with the estimated fixed effect model in dark gray and, additionally a random selection of random intercepts of the line with fixed slope. The endpoints tremor improvement and treatment response (definition above) lead to similar results and show significant differences as well.

In the analyses performed, a difference between the relationship to the center or the border of the DRT could not be shown. The estimates for the distance to the center are slightly larger than for the distance to the border. However, as the two measurements were strongly correlated, a (statistical) separation of the effects was difficult.

## Discussion

The purpose of this study was to investigate the short-term efficacy of DRT stimulation on tremor improvement. After defining our primary hypothesis that “the DRT is a common tremor target,” we have here conducted retrospective work in order to look for efficacy underpinning our hypothesis.

As far as we know, a statistically significant relationship between distances to DRT tremor improvement was not reported so far. Across different tremor origins (diseases), we found a significant relation between intraoperative tremor improvement and distance to the DRT reliably characterized by the TiCR which is an easily obtainable measure and suitable for intraoperative appraisal of stimulation efficiency and electrode position. Diffusion tensor imaging magnetic resonance imaging has emerged as a powerful tool to plan, perform, and evaluate functional neurosurgical interventions [9, 17, 54]. However, the only CE marked integrated solutions to perform these interventions rely on deterministic fiber tractography (dFT). Whether these deterministic approaches are valid in localizing complex fiber pathways of interest is a matter of debate, and there is literature emerging on this question. In fact, neuroscientists debate the whole validity of tractographic approaches and suggest other and advanced evaluation methods. With the emergence of advanced analysis tools – like spherical deconvolution and anatomically constrained tractography – problems like kissing, branching, and crossing fibers and the effects of these can likely be overcome [5, 10, 52]. However, these methods are complicated and require a level of dedication on the image processing side that might be huge obstacles to most groups, especially those with a focus on the clinical context. Moreover, neuroscientists demand *b* values of 3000 indicating a significant amount of sequence repeats in order to save a decent signal to noise ratio. This in itself prolongs the scan duration that renders it next to impossible for the average movement disorder patient unless scanning is performed under general anesthesia. A very advanced imaging sequence like the RESOLVE sequence, which takes care of deformation and other problems, demands a scanning time of up to 1.5 h, which is 3 times longer than our average scanning sequence [45].

### Stimulation efficacy relates to target structure proximity and the current employed

The proximity of a stimulation site and an effective target structure intraoperatively results in superior symptom control (Fig. 8). Specific to this work, increased proximity to the DRT (as the defined target structure) resulted in superior tremor improvement (as the target symptom) while using less current. An effective stimulation site is embedded in an electric field, which in part transforms enclosed brain into a volume of activated (brain) tissue (VAT). Within this VAT axonal structures undergo activation. The distance electric currents travel – and by this the size of an individual electric field (EF) – is among other factors dependent on the applied current amplitude. Current amplitude and EF size are quasi proportional and follow to a certain extent a linear relation [37, 49]. Simplified, the radius of the EF and the distance to a target structure can be expressed as a function of the stimulation current applied [12, 37, 49] (cf. Figs. 2 and 8). As a rule, similar symptom improvement at lower current amplitude indicates shorter distance to the target structure. Other groups have not been able to detect a linear relation during macro-stimulation [14, 55, 60]. However, they used voltage constant stimulation which is strongly related to changing impedances in the target region. We and others had previously used simple linear regression in order to find a *symptom improvement to distance relationship* for the DRT [14, 60]. However, linear regression might be a too simple model since it does not take the individual magnitude of a stimulation response into account which might be related to the disease treated or to simple and subject individual tissue properties at the stimulation site. We are aware of the more advanced models for volume of activated tissue (VAT) estimation or pathway activation modeling (PAM) [8, 25, 38] which we have not used here.

With regard to the clinical context, dFT has shown to be beneficial [3, 4, 12, 21, 22, 37, 53, 59, 60], and clinical trials are underway with the purpose to prove beneficence of DRT targeting for tremor [51, 56]. Deterministic tracking might be “accurate enough” for planning purposes in regions with little fiber crossing like the DRT, the subthalamic pyramidal tracts, or the superolateral medial forebrain bundle (slMFB) [12, 18, 37, 59]. On the other hand, we and other groups fail to reliably show a complex structure like the “crossing of the DRT” with our deterministic approach although this only plays a minor role for our stimulated part which is located distal from this crossing (*for further discussion, see limitations*). Despite these limitations, dFT has its advantages and more advanced analyzation methods for DTI sequences like probabilistic, and global tracking with their time-consuming evaluation will likely stay reserved for pre- and postoperative evaluation purposes [1, 2, 28, 34, 52]. It is of note that some groups have now started to augment dFT in planning settings with normative templates derived from different tracking modalities [53].

### Anatomical considerations regarding the cerebello-thalamo-cortical projection system and stimulation effect

Since the first mention of the DRT as a potentially tremor-reducing structure (13), there has been more detailed anatomical work on the structure [39, 41]. It was found that there are distinctive parts of the DRT that do not cross the midline and have – functionally and structurally segregated – distinct thalamic targets. Our non-decussating depiction, however, seems to be underpinned with predominant connection to VLA nucleus [44]. A parallel existence of crossing and non-crossing fibers might account for some of the side effects that occur with even unilateral long-term stimulation. It is conceivable that high frequency stimulation of the DRT is likely effective because of a modulation or masking effect of the tremor frequency which is itself introduced via a cerebellar pathology. There are some questions open. Why does a unilateral disruption of the DRT under conditions of a therapeutic intervention [11] alleviate tremor without detrimental side effects while sometimes a spontaneous lesion leaves a patient with catastrophic functional outcome [36]? One reason might be a shift of DRT physiological function to the side contralateral to the tremor. This is, however, mere speculation. Moreover, supposedly maladaptive effects can be seen as stimulation-induced cerebellar syndrome after DBS which might be related to an antidromic activation of the cerebellum [50] via DRT and other pathways. Habituation effects and also cerebellar symptoms might in part be overcome with reprogramming of the stimulation [20]. We have, however, not further followed these clearly interesting and important findings since for this contribution, we focused on short-term tremor-reducing effects.

**Fig. 7:**
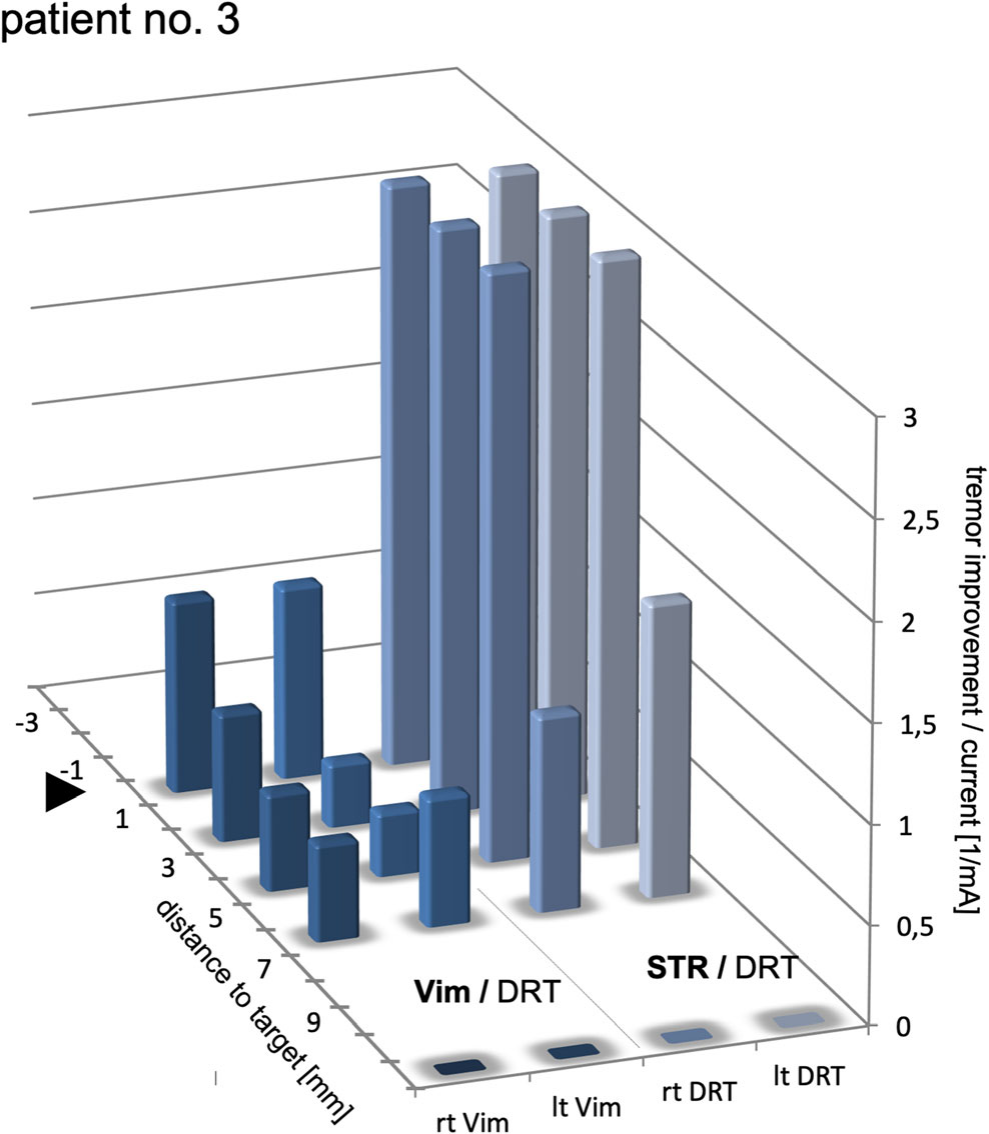
Stimulation results in a single patient (no.3). Subthalamic (STR) and thalamic (Vim) region are both tested. Note how STR results in more efficacious tremor improvement. Distances to target for both regions are shown relative for the individual target points. The STR target naturally is located 3–4 mm below MCP. Negative distance values indicate “deeper than target region”

Superior tremor improvement in deeper subthalamic positions as compared to more superficial thalamic positions (as shown in our patient 3, cf. Fig. 7) has been described before [27, 46, 47, 62] and could be due to fibers being denser in the subthalamic region before they fan out to reach more voluminous VLP/VLA (Vim) nuclei [48]. Alternatively, a more parallel stimulation (electric field lines parallel to DRT and perpendicular to electrode contact in monopolar setting) in horizontal fiber extension (cf. Fig. 2) could be the cause for better effectiveness of subthalamic stimulation: Stimulation with field lines parallel to fibers has shown in animal studies to be more activating on fibers [32] than a nonparallel stimulation.

**Fig. 8:**
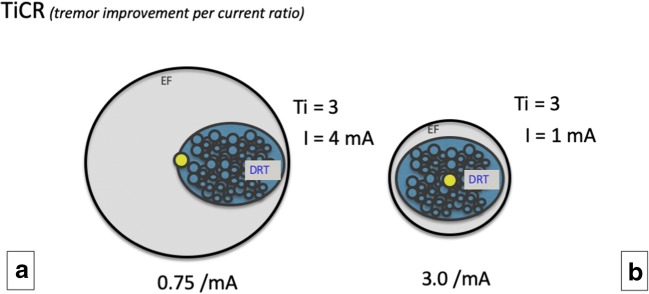
Symptom reduction to distance relationship (idealized concept). *TICR (= tremor improvement/current applied [mA]) is an efficiency measure that shows effectiveness of stimulation in correlation to the current applied.***a** and **b** show the same tremor improvement, but in **b**, only a quarter of the current is needed (1 mA) than in **a** (4 mA) because of an optimized electrode position (yellow) which allows to cover the entire DRT (blue) with less current and a smaller electric field (EF, gray)

Although there are certain limitations of our approach (see below), we think that we here provide important data that will help us to create a future prospective trial that then might serve to prove our hypothesis.

### Limitations

This work is retrospective in nature and with respect to an optimally designed trial that would prospectively search for evidence for the primary hypothesis, and the here reported data needs to be interpreted with some caution as certain limitations apply.

We do not present outcome data which are topic of separate investigations. It is, however, generally accepted that intraoperative stimulation effects are correlated to clinical outcome [58] and that these effects can be reflective of the mere distance to target white matter tracts [12, 58]. Long-term results would have been of particular interest. We cannot conclude on long-term effects of stimulation in a scientific way. Our clinical impression is that with the application of DTI tractographic planning over time, patients of this cohort showed good improvement, certainly not worse than with conventional MCP-based targeting. However, in this series, intraoperative testing was applied (besides tractographic planning) probably not so much changing electrodes’ positions compared to conventional series. The clinical efficacy of a pure tractographic approach is the focus of a currently conducted study at our institution [49].

The heterogeneous patient group with its various tremor origins is a concern since the magnitude of the effects observed is hard to appreciate between subjects anyways but more so in subjects with different tremor types. However, our statistical evaluation with the tremor effect showing a variance which is patient- and therefore disease-specific (since the tremor effect of stimulation is patient specific) might be able to circumvent this.

Our neurological raters were only incompletely blinded for the amount of stimulation applied or the position of the electrode according to the trajectory although it is hard to keep track during surgery. Intraoperatively, the rater was most of the times aware of stimulation depth but was blinded to the stimulation current applied. However, since the stimulation is typically increased in our setting with 0.5-mA increments, there was certainly some suspicion as to how the stimulation was applied. Therefore, the intraoperative rating results are potentially biased (later in the stimulation – > closer to target – > tremor improvement should be better). Tremor reduction is furthermore ranked on a subjective scale (0–3 points improvement). Possibly mechanized approaches would be more valid. In a prospective approach, an ideal setting would have been to present data points along a defined trajectory. However, because we are here doing a retrospective analysis of a case series, not all possible observations along a trajectory were measured. We are dealing with an incomplete dataset geared to clinical needs. We cannot completely rule out that these circumstances have added to the positive results of our study with respect to the primary hypothesis.

The large electrode geometry (1.3-mm diameter) might lead to some lesioning effects which influence a tremor rating. We have not observed this as an important problem. Certainly, using an electrode with a smaller geometry would significantly add to a reduction of this effect.

While one way of performing the electrophysiological analysis would be to look at the finally implanted DBS electrodes and perform testing while at the same time determining the exact contact location, we here chose a different route. Postoperative testing of patients was logistically not possible. Therefore, we had to rely on the intraoperative test protocol. This gives the advantage to look at more stimulation points along the electrode axis than with the geometry of a finally implanted DBS electrode. We used the finally implanted DBS electrodes (and their depiction in the postoperative CT scan) to draw conclusions on the actual intraoperative stimulation points. We have used intraoperative X-ray to confirm positioning of the test electrode in conjunction with the final DBS electrode positions. We felt confident to achieve a rather accurate simulation like was done successfully in previous work [12, 60]. We can however not rule out that this method introduces some error in the detection of some of the stimulation points regarded.

Limitations of the deterministic tracking have been discussed above. The anatomical crossing of the DRT as described in classical anatomy [42] cannot reliably be shown with simple tractographic methods. Newer research in human anatomy – confluent with primate anatomy – shows that the DRT in part is non-decussating [44]. However, dFT is the analysis tool that is least likely to resolve the complex fiber anatomy of this crossing. We are aware of this limitation. Other groups have analyzed the cerebello-thalamic pathways in greater detail and were able to reliably show the fiber crossing [1]. However, probabilistic tracking has not been shown to be superior on the individual level. As multiple analyses were performed, all *p* values and results have to be interpreted with caution and cannot be regarded as proof.

## Conclusion

In this uncontrolled case series, we used intraoperative test stimulation to appreciate the symptom reduction to distance relationship during tremor surgery. TiCR is a very simple measure that helps to intraoperatively appreciate stimulation efficacy. We have here for the first time found further statistical hints for DRT as a common tremor-reducing structure in the various indications – which was our primary hypothesis – and in various stereotactic targets (Vim, cZI, STA) which all seem to be grouped along this pathway. Limitations need to be regarded as this a retrospective case series. The decision to implant a DBS electrode at a given position was in this contribution finally based on the intraoperative test results. However, the DTI FT information guided the primary definition of a trajectory and also accounted for anatomical variations (in DRT). In the presented patient cohort, 90.3% of the electrodes were placed on this initially tractographically planned trajectory. Thus, tractographic methods helped to identify an individually beneficial implantation point without testing multiple trajectories, intraoperatively. This might have the potential to reduce surgery time and complication rates. The further analysis of the tractographic approach to DBS with its application in specifically designed clinical trials is the focus of our current research.

## Abbreviations

AC: anterior commissure
CT: computed tomography
DBS: deep brain stimulation
dFT: deterministic fiber tracking
DRT (or DRTT): dentato-rubro-thalamic tract
DTI: diffusion tensor magnetic resonance imaging
FT: fiber tractography
LMM: linear mixed model
MCP: mid-commissural point
MRI: magnetic resonance imaging
PAM: pathway activation modeling
PC: posterior commissure
pFT: probabilistic fiber tracking
SNr: substantia nigra pars reticulata
STA: subthalamic area
STN: subthalamic nucleus
STR: subthalamic region
TiCR: tremor improvement to current ratio
VAT: volume of activated tissue
VCA: ventralis caudalis anterior nucleus
VCP: ventralis caudalis posterior nucleus
Vim: ventral intermediate nucleus of thalamus
